# Supplementary figures and images for: Predictable motion is progressively extrapolated across temporally distinct processing stages in the human visual cortex
Source: PLoS Biol. 2025 May 23;23(5):e3003189. doi: 10.1371/journal.pbio.3003189 (PMC12133177; doi:10.1371/journal.pbio.3003189)

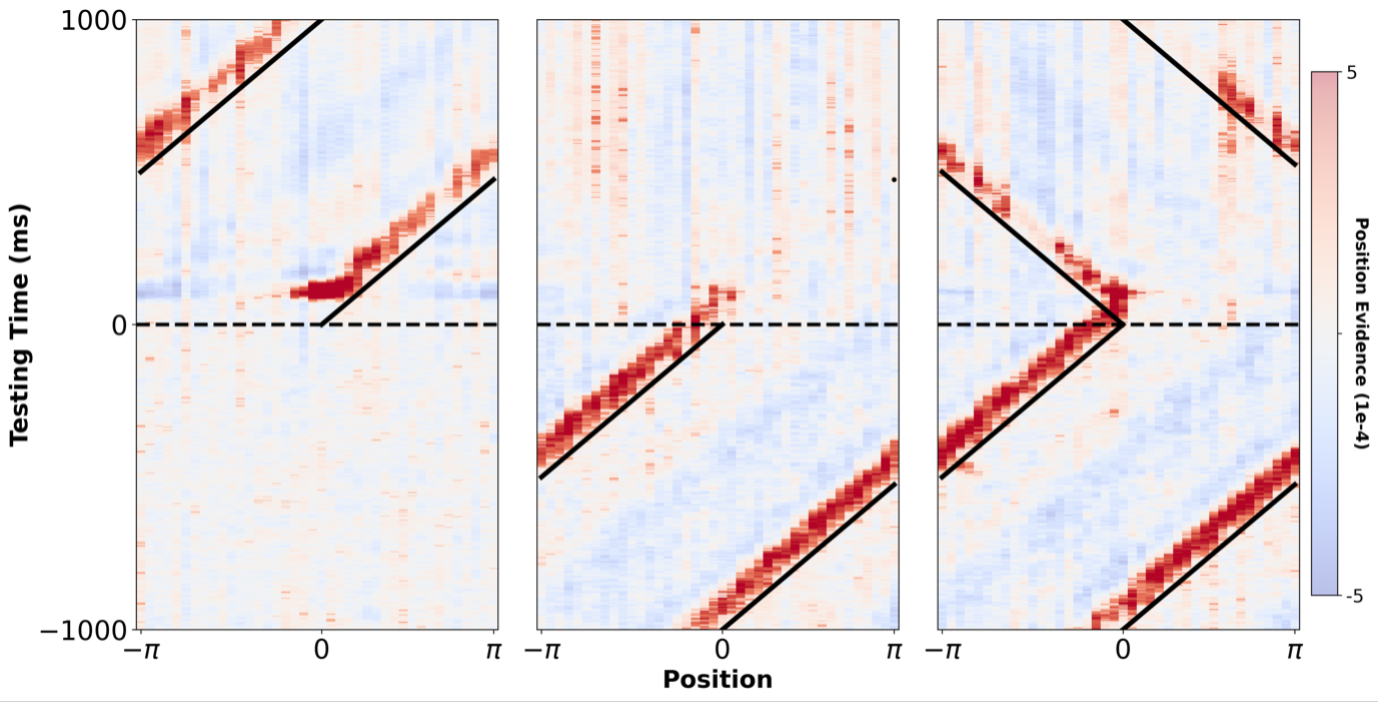

Supplement: S1 Fig — From left to right, the panels show group-level spatio-temporal maps centered around motion onset, offset, and reversal. Diagonal black lines mark the true position of the stimulus. Horizontal dashed lines mark the time of the event of interest (stimulus onset, offset, or reversal). Red indicates high probability regions and blue indicates low probability regions relative to chance (0.025). The raw data are plotted with low opacity. Semi-transparent overlays denote positions where one-sample t-tests (one-tailed) against chance were significant at thresholds of p < .05 (medium opacity) and p < .01 (full opacity), respectively. (TIFF) [file pbio.3003189.s001.tiff]

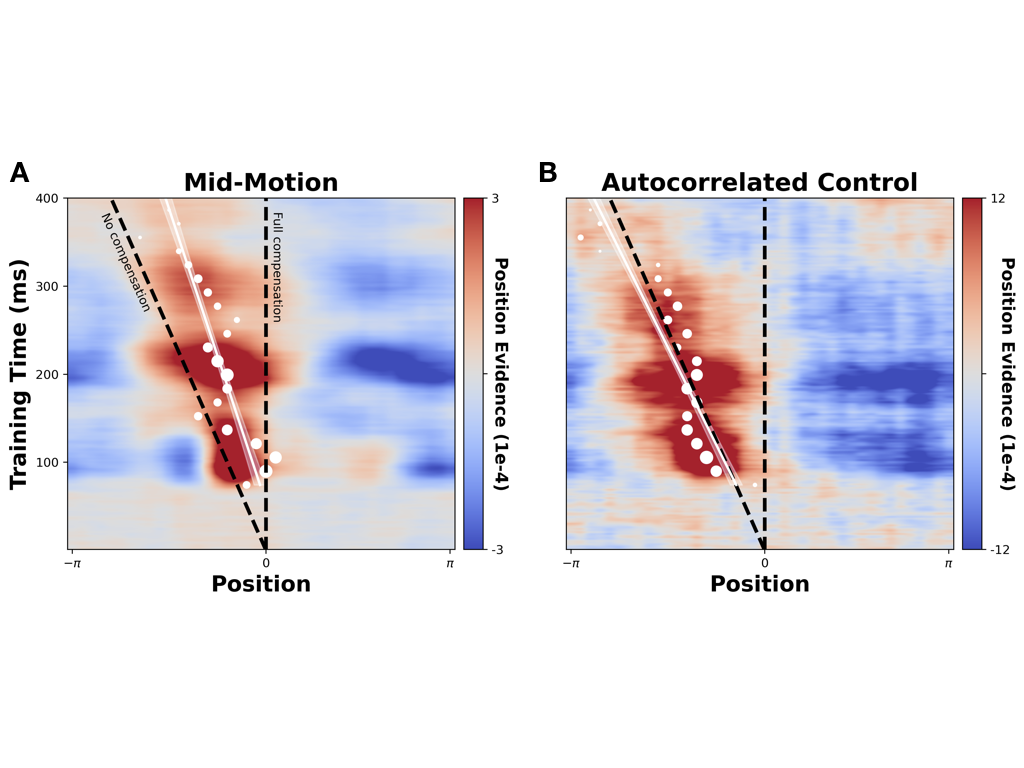

Supplement: S2 Fig — To ensure that the main latency shift effect is robust across specific analysis choices, we re-plotted Fig 4A and 4B overlaying the centroid (i.e., vector average), instead of the point of peak probability, as a discrete position estimate. Examining S2 Fig, the same effect can be observed as we report in the main text, building confidence that this does not depend on the specific read-out method we choose to employ. In the main text we use the peak probability estimate as a more conservative read-out method, since this does not display the same extrapolative properties as the centroid during early processing (see Fig 3). All plotting conventions are the same as in Fig 4. (TIFF) [file pbio.3003189.s002.tiff]

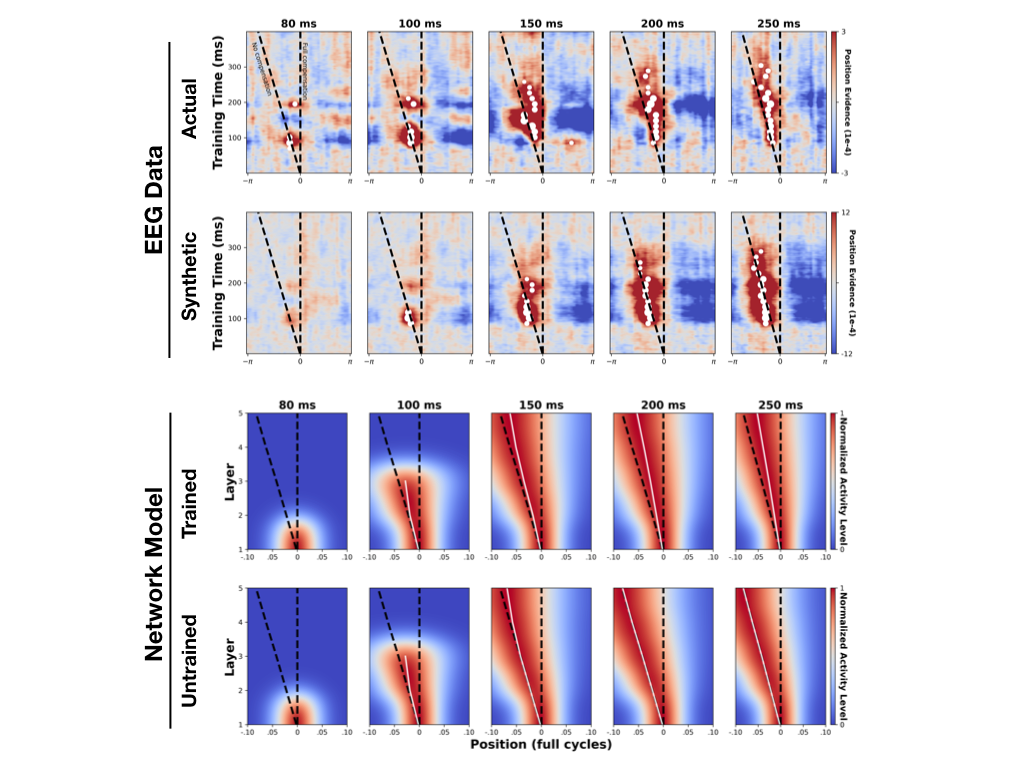

Supplement: S3 Fig — The top two rows show the temporal evolution of position information in the 250 ms following stimulus onset in the EEG data (top row: actual data, bottom row: synthetic control data). Bottom two panels show the equivalent for simulated network activity (top row: trained STDP model, bottom row: untrained control model). In the main analysis we consider the ‘steady state’ of position representations formed after sustained exposure to motion along a predictable trajectory, and found evidence of progressive extrapolation. However, after the initial appearance of a moving object, position representations must necessarily lag, since motion extrapolation is only possible once the object’s velocity has been established. Here, we conduct an additional exploratory analysis to examine how rapidly motion extrapolation arises when a moving object first appears, and how the temporal evolution of this effect may be accounted for in the STDP network model. In S3 Fig we compare how both decoded and simulated positional representations evolve over timepoints immediately following stimulus onset. Initially, the decoded maps generated from the raw and synthetic EEG data (top panels) are similar. However, from ~150 ms the bulk of the high probability region in the raw map begins to shift forwards, with only a small portion of activity left traveling diagonally along the No-Compensation line. No such shift occurs in the synthetic map, with activity remaining centered on the No-Compensation line. This indicates that it takes ~150 ms for the ‘steady state’ temporal shift which we observed after sustained exposure to smooth motion to emerge. For the simulated maps, we can see that the same forwards shift occurs in the population-level activity of the trained STDP model, but not the untrained (control) model. In the trained model peak activity initially following the No-Compensation line, but then gradually shifts forwards across later timepoints. This occurs because of the velocity estimati [file pbio.3003189.s003.tiff]
