# Supplementary material for: Predictable motion is progressively extrapolated across temporally distinct processing stages in the human visual cortex
Source: PLoS Biol. 2025 May 23;23(5):e3003189. doi: 10.1371/journal.pbio.3003189 (PMC12133177; doi:10.1371/journal.pbio.3003189)
Supplement: Attachment — Submitted filename: Turner_response_letter.pdf [file pbio.3003189.s004.pdf]

Response to Reviewers

Thank you for the opportunity to submit a revised version of our manuscript. We are grateful for the constructive feedback from both reviewers, and have extensively revised the manuscript to address their comments.

Throughout this document reviewer comments will be bolded and quoted sections from the manuscript will be italicized. For clarity and cross-referencing purposes, we have coded each of the reviewers' comments (e.g., R1C1, R1C2 etc.). Line numbers are used to indicate the location of specific changes in the revised manuscript. Figures which are included in the response letter but not in the revised manuscript are prefixed with the letter R. Figures included in the revised manuscript retain their manuscript-specific figure number. All changes in the revised manuscript have been highlighted in yellow.

Reviewer #1

The authors propose to study an important question of how the human visual system allows extrapolating visual motion cues to assist in action driven behavior. Some examples to illustrate this issue come from sports (as in baseball or tennis) when it is critical to extrapolate quickly and precisely the ball's future location in order to determine how, where and when the observer's future action is to take place. The authors indicate that their motivation comes from deficient neurophysiological (and perhaps electrophysiological?) knowledge about this topic in the human brain and to that end they examine electrophysiological EEG signals from a group of 18 human observers that view a rotating target in a predefined circular trajectory (that could however be unpredictable with respect to its rotation direction - whether clockwise or anticlockwise and switches between them). The authors apply a learning neural network on the data and propose the network performance does extremely well in localization of the location of the target. While the manuscript has beautiful figures and presents an apparently well-motivated study, there are major concerns related to study presented in the manuscript.

We thank Reviewer 1 for their thorough summary of our work and constructive feedback.

Major

R1C1). The authors suggest they are examining the ability to extrapolate target location based on motion cues. However, they may be extrapolating predominantly neural signals from the oculomotor system. The authors report they used a moving target that typically cause involuntary pursuit eye movements to follow the target. Since the authors do not report they used eye tracking in their study and do not report that they instructed the participants not to move their eyes and they do not report that they excluded data where fixation was not kept, a very likely scenario is that the eyes of the participants were mostly on the target, meaning that the stimulus was mostly in central visual field. Pursuit eye movements that likely took place most of the experiment activate the oculomotor system and therefore the neural activity measured in this study (not much has been said in the manuscript about localization of the sources of the activations recorded) could in fact reflect these oculomotor signals and not visual motion related signals. Of course there are times when there are likely catchup corrective saccades that take place, but these also involve oculomotor system activation. Did the authors examine electrodes near the FEF location? Again - since there is no report of any eye tracking taking place in the study, it is difficult to infer if the signals reflect anything to do with visual motion extrapolation, or perhaps oculomotor signals or both.

We agree that it is crucial for us to demonstrate that eye-movement-related confounds are not driving our results. First, we will explain why we are confident that this cannot explain our findings. Then we will outline the changes we have made to the manuscript to more clearly communicate this.

For three main reasons we are confident that eye-movement-related confounds cannot explain our results:

Firstly, we conducted a searchlight analysis over electrodes to determine the source of position-related information in our decoding analyses (see Fig R1A below, a duplicate of the inset in Fig 1C in the manuscript). Examining the results of this analysis we can see that decoding is being driven by activity from occipital electrodes, over the visual cortex. This is in contrast to previous work showing that eye-movement related confounds in neural decoding analyses typically manifest over frontal regions (see Fig R1B, adapted from Quax et al., 2019 – *NeuroImage*). In the results of our searchlight analysis, we see no activity over fronto-central sites. Hence, we conclude that the visual cortex is the most likely source of the signals driving our decoding.

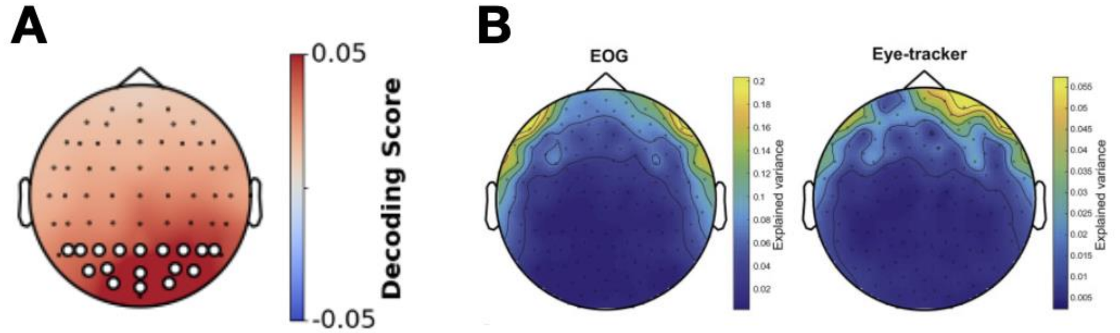

Fig R1. Comparing the source of position-specific information in our analyses with the source of eye-movement related confounds in Quax et al., 2019. **A)** A scalp map of average decoding accuracy from a search light analysis, in which position decoding was repeated at each electrode, including its immediate neighbours. Position-related information is localised over parietal/occipital electrodes, with a clear absence of decodable information at frontal sites. **B)** Both scalp maps show the variance explained by eye movements in the performance of linear decoder trained to predict the position of a visual stimulus from MEG recordings (see Quax et al. 2019 – *NeuroImage*). For both eye movement recording methods (EOG and eye-tracker), decoding confounds are most clearly expressed in frontal sites, with an absence of confounding signals at occipital recording sites.

Secondly, while we did not employ an eye tracker, we did explicitly instruct participants not to move their eyes to the stimuli. We also used a fixation target which has been shown to best reduce eye movements and promote fixation (Thaler et al., 2013 – *Vision Research*). Given that the stimuli were presented at a distance of 7.7-9.4 dva from fixation (from inner to outer edge), with the static stimuli presented at a rapid temporal rate (1 every 200 ms) and the dynamic stimuli moving at 48 dva/s, it is highly unlikely that participants would have been able to reliable fixate on the stimuli. Moreover, even if the moving stimuli occasionally triggered involuntary smooth pursuit movements, such movements will not have been triggered by the stationary localiser stimuli. Hence, it is highly unlikely for there to be a common eye-movement signal across training and test sets.

Finally, for the main LDA-based mapping analyses we restricted our focus to only parietal/occipital electrodes, and trained the decoding models only on neural activity patterns 75-125 ms after the onset of the localiser flashes. Since saccade onsets, and the resulting eye-movement-related confounds in decoding performance, have been shown to occur most often after >200 ms (see Quax et al., 2019 – *NeuroImage*), this further reduces the likelihood of eye-movement confounds explaining our results.

In sum, given the occipital source of position-specific information in our neural recordings, and the fact that we restricted our main analyses to these occipital sites (directly over visual cortex) at a very early time window (75-125 ms), we believe it is extremely unlikely that our results can be explained by eye-movement related confounds. Instead, the most likely account of our analyses is that they are tapping into the initial feedforward sweep of visual information processing, and the position-specific activity patterns evoked by our stimuli in retinotopically organised regions of visual cortex.

In the Discussion of the revised manuscript we now explicitly consider and rule out this potential confound as follows (Lines 536-552):

'Finally, it is important to consider the possibility that the observed effects were driven by eye-movement related confounds. For three main reasons we are confident this is not the case. Firstly, the results of our searchlight analysis (Fig. 1C inset) demonstrate that decoding is being driven by activity from occipital electrodes, over the visual cortex. This is in contrast to previous work showing that eye-movement related confounds in neural decoding analyses typically manifest over frontal regions (see Fig. 6 in [47]). Secondly, while we did not employ an eye tracker, we explicitly instructed participants not to move their eyes to the stimuli and used a fixation target which has been shown to best reduce eye movements and promote fixation[48]. Given that the stimuli were presented at a distance of 7.7-9.4 dva from fixation (from inner to outer edge), with the static stimuli presented at a rapid temporal rate (5 Hz) and the dynamic stimuli moving at 48 dva/s, it is unlikely that participants would have been able to continually and reliable fixate on the stimuli. Finally, for the main LDA-based mapping analyses we restricted our focus to only occipital electrodes, and trained the decoding models only on neural activity patterns 75-125 ms after the onset of the localiser flashes. Since saccade onsets, and the resulting eye-movement-related confounds in decoding performance, typically occur after >200 ms[47], this further reduces the likelihood of eye-movement confounds explaining our results.'

R1C2) Motion-sensitive regions as LGN, V1, and MT/V5 have a retinotopic organization so when a stimulus appears in one hemifield (e.g. right or left) the contra-lateral brain regions are activated. But, since the stimulus in this study is likely to have fallen in central vision most of the time (no eye tracking seems to have taken place to remove data when eyes did not fixate, and pursuits likely to have dominated eye position during the experiments) - it is hard to assume that the decoding data is based on visual cortex retinotopic information apart from central visual field stimulation.

We respectfully but strongly disagree with this suggestion. Given the reasons outlined above (see our response to R1C1), it is highly unlikely that the stimuli will have been in participants' central vision most of the time. The searchlight analysis makes it clear that our findings arise due to position-specific differences in electrical activity recorded at posterior electrode sites. The clearest explanation of these findings is that subtle differences in electrical activity arise from the retinotopic organisation of the underlying neural populations, where changes in the position of an object lead to changes in the evoked activity pattern, and thus multivariate changes in activity across occipital electrodes.

R1C3) The Methods section and statistics in general are surprisingly lacking many important details that are essential to substantiate the presented scientific findings. Some examples (by all means this is a very incompletely list of missing information):

Thank you for highlighting aspects of the manuscript which were lacking in detail. Below we outline the changes we have made in light of these points, to improve the overall clarity of the manuscript.

R1C3)a. It is unclear which data are presented in each figure. Are the data based on one participant, on all or on a group of participants, are they based on one or all or a group of the electrodes (which of them?). What are the means and what is the variance in the data. Hardly no descriptive information is provided about the data. Data in Figures 1, 2, 3, 4 are unclear about these issues.

In the captions to Figs 1-4 we now specify that these analyses were conducted at the group-level (i.e. using the data from all participants). For the initial SVR-based analyses, we now specify that data from all electrodes were used. For the searchlight analysis, we iterated across electrodes, taking each electrode plus its immediate neighbours – we note this in the figure caption. Given that the searchlight analysis revealed strong position information decoding over the occipital cortex, for all subsequent LDA-based mapping analyses, only data from the 17 posterior-occipital electrode sites were used. We note that this sub-setting is a common procedure, adopted in past studies (e.g., Hajonides et al., 2021 – *NeuroImage*; Wolff et al., 2017 – *Nature Neuroscience*). These electrodes are highlighted in the inset in Fig 1C. In addition to noting this information in the figure caption, we now also highlight it in the Method (Lines 692-694):

'For these analyses, we restricted our focus to the data recorded from the 17 occipital parietal electrodes (P7, P5, P3, P1, Pz, P2, P4, P6, P8, PO7, PO3, POz, PO4, PO8, O1, Oz and O2; see Fig 1C inset).'

R1C3)b. Hardly no statistics are provided to support the validity of the findings (of the decoded data, of the presented results, even of the statistical maps). Even for cluster correction - the authors do not indicate how was this cluster correction achieved, what was the threshold, size of the cluster used to correct for any results.

Details of the cluster correction procedure (cluster forming threshold, critical alpha level, number of permutations, and MNE function used) are given in the statistical analyses section of the Method (Lines 709-710). Given that this was not readily apparent, we now also list the cluster forming threshold and number of permutations when first mentioning the cluster-corrected thresholding (see Fig 1 caption).

All analyses in Fig 1, as well as the key finding of an accelerated emergence of position representations in Fig 4, are supported by statistical tests. However, the question of statistically thresholding the probabilistic maps in Fig 2 is in fact rather complex. In the initial draft of the manuscript we deliberately restricted our focus to exploring qualitative patterns in these data. This was due to the fact that the spatio-temporal maps have somewhat unique dependencies, making them sub-optimal for traditional approaches like 2D cluster-based thresholding (such approaches are overly conservative). Indeed, since at any given time point the *entire* distribution encodes spatial information, statistically thresholding sub-sections of the distribution is not a common procedure (e.g., see distributions presented in Hajonides et al., 2021 – *NeuroImage*; Wolff et al., 2017 – *Nature Neuroscience*; Turner et al., 2023 – *Journal of Neuroscience*). Instead, in experiments involving static stimuli, it is common to convolve a cosine function with the distribution. This gives a single estimate of spatial tuning across the entire distribution, and statistical testing of the convolution results across time can be conducted (e.g., Hajonides et al., 2021 – *NeuroImage*, see their Fig 2C). Unfortunately however, in the present context, with a dynamic stimulus, this approach is also sub-optimal since the decoded distributions become asymmetric after ongoing exposure to predictable motion (e.g., see our Fig 3B). Nevertheless, despite these considerations, and in the interest of clarity, in the revised manuscript we now include an additional supplementary item (Fig S1), showing a statistically thresholded version of the spatiotemporal maps. Given the above considerations, we have opted for the simple approach of conducting uncorrected one-sample t-tests (one-tailed) with thresholds of p <

.05 and p < .01, adding transparent overlays of increasing opacity for each significance level. This allows us to visualise spatiotemporal locations where position evidence is significantly greater than chance. Given that statistical thresholding of probabilistic maps is somewhat non-standard, we have opted to retain the un-thresholded maps in the main manuscript, with the thresholded maps included as a supplement (Fig S1).

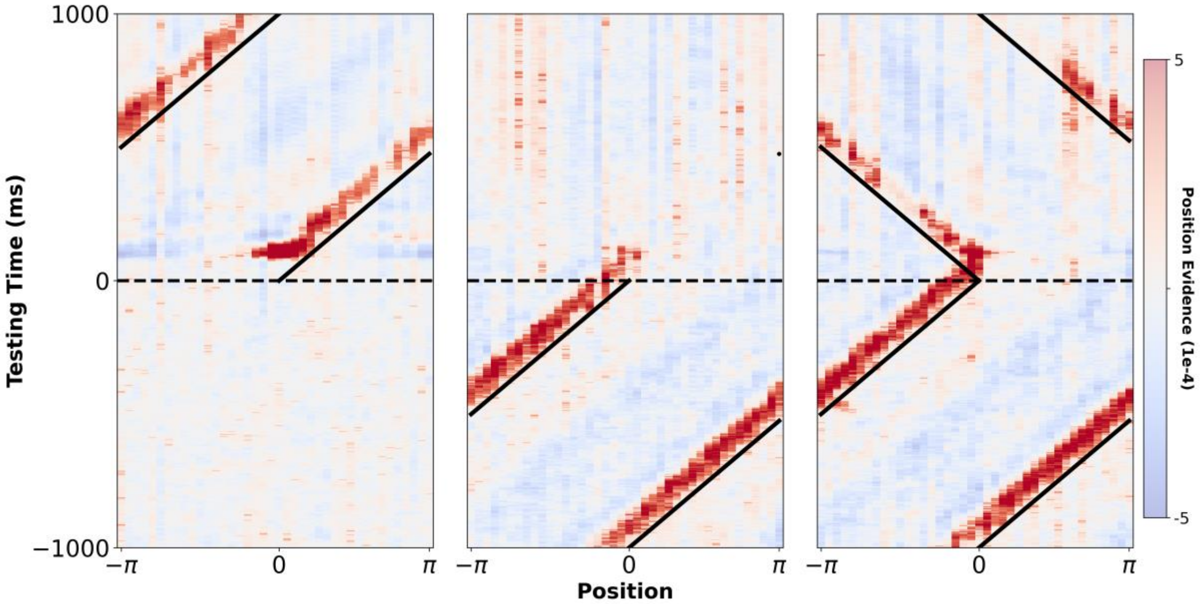

Fig S1. Statistically thresholded probabilistic maps. From left to right, the panels show group-level spatio-temporal maps centred around motion onset, offset, and reversal. Diagonal black lines mark the true position of the stimulus. Horizontal dashed lines mark the time of the event of interest (stimulus onset, offset, or reversal). Red indicates high probability regions and blue indicates low probability regions relative to chance (0.025). The raw data are plotted with low opacity. Semi-transparent overlays denote positions where one-sample t-tests (one-tailed) against chance were significant at thresholds of p < .05 (medium opacity) and p < .01 (full opacity) respectively.

We have also expanded the statistical analyses section of the Method to explain the statistical tests we have conducted in greater detail (Lines 707-715):

'For the SVR-based decoding analyses (Fig 1), we performed cluster-corrected one sample t-tests (two-tailed) against zero using a critical alpha level of .01 and a cluster forming t-threshold corresponding to a p-value of .01 (computed via the MNE function 'spatio_temporal_cluster_1samp_test', with 2^{12} permutations). For the statistical thresholding of the LDA-derived probabilistic maps (see Supplement 1), we conducted one-sample t-tests (one-tailed) at thresholds of p < .05 and p < .01. To statistically test for a difference in the slopes of the peak-position estimates from the actual and synthetic datasets in Fig 4, we fit a linear regression model to the group-level data. We predicted the peak position estimate from training timepoint and data type as well as their interaction.'

Finally, we note that the analyses presented in Fig 3 are meant as exploratory, qualitative analyses. Their main purpose is to raise a speculative possibility as to how a discrete read-out of stimulus position may be achieved from the full probability distribution, and to invite further empirical investigation of this (e.g., via a speed manipulation experiment). As such we do not believe it is necessary to conduct post-hoc statistical testing here. In the revised manuscript we explicitly stress the qualitative and speculative nature of these analyses as follows:

'To qualitatively examine the shape and precision of the decoded probability distributions…' (Lines 194-195)

'Considering Fig 3A&B, an important auxiliary question arises…' (Line 212)

'For consideration of how this speculative proposal may be quantitatively tested, see the Discussion' (Lines 231-232)

Finally, we have re-titled Fig 3, to more clearly convey the qualitative nature of these analyses: *'Qualitatively examining the shape and potential discrete read-out of decoded probability distributions'* (Lines 233-234).

R1C3)c. Not indicated what measure error bars represent in each panel they appear. Many more details are missing as well.

The shaded error bar in Fig 4A&B is labelled as denoting the standard error of the mean in the figure caption. We now also note that the error bars in Fig 1C&D represent the standard error of the mean.

Finally, we note that all of our analysis code and accompanying data are openly available, allowing readers to re-create all analyses and visualisations, and scrutinise all fine grained analysis choices: https://github.com/bootstrapbill/neural-location-decoding

R1C4) The authors report that they used a 64-channel EEG cap, so it is essential to provide the electrodes that the data was based on. Since this is looking into motion-based brain analysis it is odd that no localization or confinement of the analysis is performed to electrodes that are typically close to the motion-dedicated region/complex of human MT/V5+ (e.g. see Vilhelmsen et al, 2015 with P3 and P4 as electrodes of interest and there are many additional such examples) which is known to be very close to the skull so likely very good SNR arising from it.

As mentioned above (see R1C3a) all electrodes were used in the SVR-based analyses, and the 17 most parietal/occipital electrodes were used in the LDA-based mapping analyses. This is now more clearly specified in the text of the manuscript. The confined set of electrodes used for the LDA mapping includes those highlighted by Reviewer 1 (P3 and P4). This sub-selection of electrodes was done with the intention of improving the SNR of our position-decoding analyses, by restricting our analyses only to sites over visual regions (following Hajonides et al., 2021 – *NeuroImage* & Wolff et al. 2017 – *Nature Neuroscience*).

We now make this explicit in the Method:

'For these analyses, we restricted our focus to the data recorded from the 17 most occipital parietal electrodes (P7, P5, P3, P1, Pz, P2, P4, P6, P8, PO7, PO3, POz, PO4, PO8, O1, Oz and O2; see Fig 1C inset).'

R1C5) So much literature is missing that indicates that human visual cortex is involved in extrapolating visual information that it is really hard to provide a decent survey. Some examples include apparent motion in visual cortex, and additional topic. Here are just some of these: Field and Wann, 2005; Larsen et al, 2006; Sterzer et al, 2006; Ilg, 2008; Roach et al, 2011; Akselrod et al, 2014; Bosco et al, 2015; Schellekens et al. 2016.

We thank the reviewer for drawing our attention to this literature. We now cite the reviews by Ilg (2008) and Bosco et al. (2015) as evidence of putative extrapolation effects in MT/MST and downstream motor regions (Lines 64-65). We now also cite the studies by Schellekens et al. 2016, Akselrod et al., 2014, Sterzer et al, 2006, Larsen et al. 2006, and Field & Wann, 2005 as additional examples of empirical human fMRI studies which have observed motion prediction effects (Line 75).

We note that, in determining the relevant scope for the present study, we consciously restricted our focus to papers which provide evidence of motion prediction/extrapolation effects on the neural level. While the study by Roach et al. (2011) presents compelling behavioural evidence of visual extrapolation, it provides no data as to the neural origin of these effects. As such, it is outside the relevant scope of this paper.

R1C6) The question that apparently motivated this study is somewhat already answered (explanation follows). Thus, in effect the results reported here are maybe interesting at the ANN level but are much less novel in revealing novel findings about the human visual system. Explanation - from lesion studies (as LM, the motion blind patient) - it is clear that while the retina and even V1 are completely spared, MT's lesion causes loss of not only visual motion perception but also of motion prediction. Thus, it is evident that MT is critical for visual motion perception and also for visual motion prediction. In addition, motion perception can be induced by non-retinal inputs (as epileptic seizures reported in the literature, e.g. by Leff et al. and more reports) indicating that motion prediction cannot in any way be solved solely by the retina. Let alone motion perception modulations by intracranial stimulation (e.g. Zohary et al. 1994) again indicating that the brain definitely plays a critical role in determining visual motion perception which is a prerequisite for visual motion extrapolation.

We agree that there is considerable evidence for the involvement of cortical regions in determining visual motion perception, as well as some emerging evidence of putative motion prediction effects in such regions. Nevertheless, we disagree that this already answers the question which motivated the current study.

While the evidence cited by Reviewer 1 makes is clear that the brain plays an important role in the perceptual or behavioural *read-out* of motion signals, it does not address the question of where within the system motion prediction actually occurs. In the Introduction (Lines 66-72) we make this point as follows:

'…it is unclear to what degree neurons downstream from the retina simply inherit their extrapolated activity profiles, without actively driving further extrapolation. … As such, an important open question is whether neural motion extrapolation is a multi-stage phenomenon, that occurs during later-stage cortical processing.'

Neuropsychological cases like LM unfortunately do not resolve this uncertainty. Given the data from LM alone, we cannot know whether deficits in motion prediction/perception are due to the dysfunction of MT-based motion predictions mechanisms, or simply due to the interrupted signalling of motion predictions generated in lower-level regions, which must pass through MT for effective readout by downstream regions.

The fact that epileptic seizures/intracranial stimulation can generate the perception of motion is interesting, and tells us something about the role that cortical regions play in generating the perceptual experience of motion. But this does not imply that such regions are necessary for motion *extrapolation*. Given the strong evidence that perceptual experience of motion and position arises

postdictively (cf. Eagleman & Sejnowski, 2000 – *Science*; Herzog et al. 2020 – *TICS*), it's more likely that pre-perceptual mechanisms facilitate motion extrapolation, allowing for real-time interaction with moving objects. As such, from existing evidence alone we cannot conclude anything about the precise neural locus of such processes. Given these points, we stand by the assertion that it's unclear whether neural motion extrapolation is a multi-stage phenomenon, which also occurs during later-stage cortical processing. Only by simultaneously recording activity from multiple regions, and looking for cumulative shifts in predictive dynamics across processing stages, can we ascertain whether multi-stage extrapolation is occurring.

We have now expanded our discussion of this point in the Introduction, referring to the lesion evidence raised by the reviewer, and more clearly outlining the motivation for this study (Lines 66-72):

'… it is unclear to what degree neurons downstream from the retina simply inherit their extrapolated activity profiles, without actively driving further extrapolation. For example, while lesions to MT have been shown to diminish accuracy in motion prediction tasks, it is unclear whether this is caused by a disruption of predictive mechanisms occurring within MT or to interrupted signalling of predictive information generated in earlier regions. As such, an important open question is whether neural motion extrapolation is a multi-stage phenomenon, that occurs during later-stage cortical processing.'

Minor

R1C7) As there are many types of motion perception (biological, optic flow, translation, rotational, first order vs second order, object from motion, and many more) that have been shown to be supported by dedicated and yet dissociated brain mechanisms, the authors should acknowledge in their manuscript that their study involves only one type of visual motion stimulation and therefore the results may be specific and not generalize to other types of visual motions that may each potentially rely on different types of mechanisms (eventually also affecting their future extrapolations).

We agree and now note this in the Discussion as follows (Lines 516-519):

'Moreover, it is worth noting that our focus has been on smooth, first-order motion, but future studies should examine whether the current observations hold for alternative forms of motion (e.g., second order motion, biological motion, or optic flow), which potentially rely on processing mechanisms which are, at least partially, distinct.'

Reviewer #2

The authors provide experimental evidence for hierarchical extrapolation of predictable motion. A decoding scheme is used to infer the position of an unpredictable static stimulus, which is then applied to a moving stimulus. It is shown that when using the peak of the distribution, the delay is partially compensated, especially when the full distribution of position evidence is used. A biomimetic model using STDP, based on previous theoretical work, predicts these results and provides support that the connectivity is motion-based.

The paper addresses an important problem and provides novel and convincing results. However, there are many possible caveats, and it would be fruitful to address the following points:

We thank Reviewer 2 for their positive appraisal of our work and constructive comments and suggestions.

MAJOR

RC1) The decoding paradigm provides an estimated probability distribution, but any error in the model could generate patterns that are related to the model and not to the way local activity is structured. In particular, could you explain why you chose SVR and then LDA, or why you did not use logistic regression? Could you compare the efficiency of each decoding model?

Thank you for requesting clarification on this point – re-reading the manuscript we can see how the logic behind the switch from SVR- to LDA-based decoding was unclear and potentially confusing. Below we explain our reasoning, with reference to the relative efficiency of each model in its given application.

For the initial analyses (Fig 1), the aim was to search for and characterise sources of stimulus-position information within the neural signal, which could then be leveraged for a subsequent mapping analysis. In doing so, we sought to use an approach which would be maximally sensitive to any sources of information, while also being computationally efficient. Following King, Pescetelli, & Dehaene (2016) we chose to perform circular SVR. This approach respects the circular nature of the outcome variable (angular position), and allows us to efficiently derive a *continuous* measure of prediction accuracy (inverse angular error). In theory we could have used LDA for this analysis, however to avoid overly harsh model evaluation we would need to adopt more computationally intensive procedures (i.e. 'out of the box' this is a discrete prediction algorithm, with prediction accuracy tied to correctly determining class membership in a 1/40 classification problem). For example, we would have to generate a probabilistic map of the localiser stimuli and then convolve a cosine function with these maps to derive an estimate of position tuning (e.g., Hajonides et al., 2021 - *NeuroImage*). In theory this would be ok for generating Fig 1C&B, but for the time-generalised and frequency-specific analyses (Fig 1E-G) this would rapidly become computationally costly and unwieldy (i.e. we would need to repeat this process for each pixel within those subplots). Hence, for the sake of computational efficiency, and to maximise our chance of finding decodable position information in these initial examinations, we chose to use angular SVR.

For the mapping analyses (Fig 2-4), the benefit of switching to use LDA is that it allows us to generate a probabilistic prediction for each possible position simultaneously. These are crucial for allowing us to create a continuous probabilistic map. In theory, we could have used SVR or logistic regression to attempt to conduct the same analysis (e.g., by counting the frequency with which positions are mis-classified, and plotting the misclassification frequencies in a map, see King & Wyart, 2021 – *Journal of*

Neuroscience). However, this is sub-optimal as at each time point only a single prediction (as opposed to a prediction for each class) is generated, making it a much less efficient use of the data.

To aid the readers understanding of these specific analytic choices, we now explain this logic in the Method (Lines 630-656):

'Below we outline the details of the two decoding approaches we have employed, using support vector regression (SVR) and linear discriminant analysis (LDA) models respectively. Prior to this, however, we will briefly outline the goals behind each of these approaches, and logic behind choosing these specific models.

For our initial analyses (Fig 1), the aim was to search for and characterise sources of stimulus-position information within the neural signal, which could then be leveraged for a subsequent mapping analysis. In doing so, we sought to use an approach which would be maximally sensitive to any sources of information, while also being computationally efficient. Following King, Pescetelli, & Dehaene[29] we chose to perform circular SVR. This approach respects the circular nature of the outcome variable, and allows us to efficiently derive a continuous measure of prediction accuracy (inverse angular error). In theory we could have used LDA, however to avoid overly harsh model evaluation we would need to adopt more computationally intensive procedures (i.e. 'out of the box' this is a discrete prediction algorithm, with prediction accuracy tied to correctly determining class membership in a 1/40 classification problem). For example, we would have to generate a probabilistic map of the localiser stimuli and then convolve a cosine function with these maps to derive an estimate of position tuning (e.g., [51]). For the time-generalised and frequency-specific analyses (Fig 1E-G) this would rapidly become computationally costly and unwieldy (i.e., we would need to repeat this process for each pixel within those subplots). Hence, for the sake of computational efficiency, we chose to use angular SVR for these analyses.

For the mapping analyses (Figs 2-4), the benefit of switching to use LDA is that it allows us to generate a probabilistic prediction for each possible position simultaneously. In theory, we could have used SVR or logistic regression to attempt to conduct the same analysis (e.g., by counting the frequency with which positions are mis-classified, and plotting the mis-classification frequencies in a map, see [32]). However, this is sub-optimal as at each time point only a single prediction (as opposed to a prediction for each class) is generated, making it a less efficient use of the data.'

R2C2) What if you trained your model on the neural activity generated by the moving dot to predict both position *and* direction (CW or CCW)? Wouldn't you then be able to detect position accurately?

We thank the Reviewer for this suggestion. If the goal was simply to derive a real-time estimate of the stimulus' position, then we agree that this could be a useful approach. However, since our goal was to get an unbiased estimate of the degree to which position representations change under predictable conditions – that is, whether there is a predictive/extrapolatory shift during smooth motion – this approach is sub-optimal. This is because, if we train on motion-evoked signals, we would be training on data which may already include a predictive shift. Moreover, training on motion-evoked responses may lead to the training data including inverted representations for positions which were recently occupied by the stimulus. This would significantly muddy the interpretation of the probabilistic spatiotemporal maps. As such, to avoid these potential confounds, the cleanest solution is use a bank of pre-trained static position representations to generate maps from the responses to moving objects which we can be confident are unbiased by these factors. The fact that this decoding approach works, adds to a small but growing body of evidence (e.g., Johnson et al., 2023 – *eLife*) that generalisation from static flashes to moving stimuli is a viable method for tracking position-specific neural activity patterns, which avoids problematic motion-induced confounds.

R2C3) In the temporal generalisation paradigm, you observe peri-diagonal rebounds - these seem to occur with a delay of about 80ms, which is compatible both with the sensory delay and with alpha rhythms. Is this a sign of higher level feedback? Is this a rhythmic effect? For example, do you observe different power depending on the phase of the alpha wave (as observed behaviorally by Dugué or Van Rullen) or depending on the level of higher-level activity?

Thanks for raising this interesting point. One plausible interpretation of this pattern is indeed that higher-level feedback briefly re-activates lower-level position representations. However, we note that precise interpretation of the dynamic patterns within temporal generalisation matrices is notoriously difficult, with recent work showing that many different complex patterns of generalisation (some qualitatively similar to those we observe) may arise from purely feed-forward architectures (King & Wyart, 2021 – *Journal of Neuroscience*). Crucially however, since the localiser stimuli in the current study were presented at a harmonic of alpha (5 Hz) our ability to disentangle whether this is an endogenously or exogenously driven effect is limited. Given this, we feel that follow-up analyses probing this effect, at least in the current dataset, are not appropriate. However, we note that we have recently conducted related work (Turner et al., 2023 – *Journal of Neuroscience*) in which we varied the temporal rate of the stimulus and found evidence that oscillations in this same frequency range (alpha/low-beta) may indeed carry predictive position information.

Overall, we agree that this is an interesting feature of the current findings, and now highlight this as a potential target for future investigation. Specifically, we now consider this point when highlighting potential directions for future modelling work (Lines 519-535):

'… Finally, a remarkable feature of the current model (and[38,39]) is that spontaneous motion extrapolation is achieved during purely feed-forward processing. However, future studies should consider further developing these models, building in recurrent and/or horizontal connections. These may act as putative mechanisms for fine-tuning the magnitude of STDP-driven extrapolation in a delay-dependent fashion (i.e. calibrating the degree of shift to a given delay). Of potential relevance here is the fact that we see oscillatory patterns of peri-diagonal rebounds in the temporal generalisation matrix (Fig 1E), with a frequency-specific decoding analysis showing that position information is predominantly localised in the alpha/low-beta range (Fig 1F). This is consistent with recent work from our lab showing evidence that oscillations in this same range carry predictive position information[19], in line with theoretical work from Alamia and VanRullen[34] suggesting that such oscillations may be a spectral signature of hierarchical predictive coding. However, since the localiser stimuli in the current study were presented at a harmonic of alpha (5 Hz), it is difficult to disentangle the degree to which these effects are endogenously or exogenously driven. As such, examining the potential oscillatory underpinnings of these effects, and using these dynamics to constrain/determine the possible underlying neuro-computational architectures (see[32,46]) remains an exciting avenue for future research.'

R2C4) The peak of the distribution seems to be delayed while the median is aligned, showing that probability distributions are more versatile than analog values. What are possible mechanisms? A suppressive traveling wave as observed in monkeys VSDI?

Thanks for raising this interesting point. Given that we are operating at the level of decoded probability distributions, the potential underlying neural mechanisms remain somewhat open in light of the current findings. However, we agree that suppressive travelling waves, as observed by Chemla

and colleagues (2019), are plausibly involved. As such, we have expanded the Discussion to highlight this point, with recommendations for future research (Lines 484-491):

'Relatedly, future studies should also attempt to determine the specific neural mechanisms underlying the observed distribution shift. Specifically, studies could examine the potential roles of laterally spreading activity[4,6], suppressive travelling waves[45], and related mechanisms (for a recent review see[2]), in dampening activity at the trailing edge and/or enhancing activity at the leading edge of a moving stimulus, driving a distribution shift. For instance, it may be interesting to examine the degree to which anticipatory effects are abolished during visual midline transitions, as laterally spreading activation will be disrupted, with signals instead passing through the corpus callosum (e.g., see[10]).'

R2C5) - The motion reversal experiment is not fully discussed, especially at the moment of reversal one might expect an overshoot, while one observes a rather continuous prediction for the motion, as for example in the theoretical results of:
https://journals.plos.org/ploscompbiol/article?id=10.1371/journal.pcbi.1005068
- do you observe a similar effect in your model? How different are these models?

We thank the reviewer for this question. Certain details of our STDP model make it difficult to confidently simulate activity profiles surrounding motion reversals in a meaningful way. Specifically, a key feature of our model is that it gradually homes in on velocity (narrowing down a Gaussian around the detected stimulus speed). Following a sudden reversal the velocity will abruptly jump to a completely distinct value. We currently have no good theoretical basis for how that shift from one velocity to the other would be registered and encoded. This affords us with too many essentially arbitrary assumptions to be able to say something meaningful and principled about activity surrounding reversals at this stage. In a sense there is a parallel here with Khoei and colleagues (2017) modelling work, referenced by the reviewer. Their model can indeed predict a continuous motion trajectory around reversal (i.e. no overshoot and reset). However, this relies on the somewhat arbitrary assumption of an additional smoothing operation, to remove the overshoot which is otherwise present (see their Fig 6). Note, in their case they had good reason to do that, since this allowed them to capture the fact that motion reversals appear to be continuous on the conscious perceptual level (see Eagleman & Sejnowski, 2000 – *Science*; Whitney & Murakami, 1998 – *Nature Neuroscience*). In our case, since we simply aim to relate our model to the observed neural dynamics, and given that we could in principle simulate both the presence and absence of an overshoot depending on the assumptions we make, we feel that modelling this aspect of the data is premature. More neural recordings should first be collected across a range of velocities (and other parameter manipulations) to properly constrain possible accounts.

Regarding the question of differences between the present model and Khoei and colleagues model: one important difference is that the present model is, in a sense, 'model free'. That is, it contains no explicit internal model of motion dynamics. In contrast, Khoei and colleagues model includes an internal model of motion dynamics (i.e. the physical laws of motion), making it equivalent to a form of recursive Bayesian estimator (e.g., a Kalman filter). Second, while the Khoei model shows how pre-specified asymmetries in connectivity can produce motion extrapolation, the current STDP model goes beyond this, showing how these asymmetries may spontaneously emerge in an unsupervised fashion, via a simple synaptic learning rule.

Finally, more broadly, we note that the question of whether or not we observe evidence of an overshoot, ultimately boils down to the question of how downstream regions read out position representations from a given stage of processing. At the earliest stages of processing, Schwartz and colleagues (2007) observed that the peak of retinal ganglion cell activity overshoots following motion

reversal (see Fig R3 below). So why do they see an overshoot, while we do not? First, it's important to remember that we are operating at the level of decoded probability distributions, so we cannot make any confident assertions about whether or not the underlying peak of neural activity also overshoots in cortical regions. However, one possibility is that transformations are taking place during information transition from retina to cortex, changing how positional information is encoded. Indeed, such transformations must eventually take place, given that no overshoot is perceived on the conscious perceptual level (e.g., Eagleman & Sejnowski, 2000 – *Science*). In our opinion, one unique contribution that the current study makes is in demonstrating that neural activity during early cortical processing encodes a probability distribution which is distributed in such a way as to allow for (at least) two distinct position estimates to be generated: one which is predictive yet susceptible to overshoot (centroid), and one which is delayed but resistant to overshoot (peak).

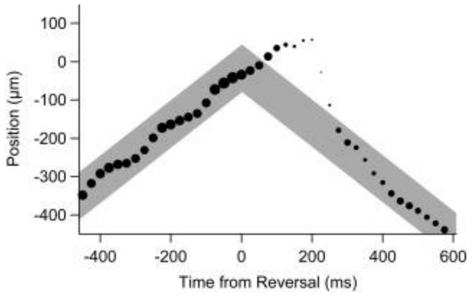

Fig R3. A subsection of Fig 6B from Schwartz and colleagues (2007). Location of the peak of the neural image (circles) plotted as a function of time from reversal; size of circles represents the peak firing rate; location of moving bar through time is depicted by the shaded region.

In light of the points above, we have significantly expanded our discussion of the dynamics surrounding motion reversal (Lines 457-483):

'… Considering the observed changes, a hypothesis which emerges is that the shape of this distribution is modulated in such a way as to allow different point estimates of a moving object's location to be read out, depending on current task demands. Specifically, if accurate real-time interaction is paramount, then an estimate of the stimulus' real-time position may be derived from the centroid of the distribution. However, when the stimulus disappears or changes trajectory this can overshoot and be unreliable. Conversely, when real-time interaction is less important, and accurate trajectory perception is relatively more important, the peak of the distribution may be taken, yielding a more stable, but delayed, estimate of the stimulus' position (i.e. it reliably tells you the stimulus' recent position). In other words, at any given moment, different estimators may be used, either to determine the recent position of a stimulus with high reliability (peak), or the likely current position of a stimulus, with lower reliability (centroid). Here we note related observations made by Schwartz and colleagues[7] who observed that the peak of population-level retinal ganglion cell activity overshoots following motion reversal (see their Fig 6B). In this study, we are operating at the level of decoded probability distributions, so cannot confidently assert anything about the underlying distribution of neural activity. Nevertheless, one possibility is that transformations occur as information passes along the visual hierarchy, gradually removing the retinal overshoot, eventually aligning with observations of smooth reversal trajectories on the conscious perception level (see [41-44]). To further test whether such transformations are taking place, and whether early cortical regions encode a positional probability distribution from which different discrete estimates can be read out according to task demands, future studies will need to vary the speed of the moving stimulus. The central test will be whether the distribution centroid shifts to align with the real-time position of the stimulus regardless of its speed, serving as an effective real-time estimate. If this occurs, then future studies would also need to determine whether/how downstream regions can extract summary measures (such as a centroid) of

population-level activity in early visual regions, and ultimately how these relate to behaviour (such as reaching or related targeting actions).'

MINOR

R2C6) Figure 1: a decoding score of 0.05 seems significantly different from 0, yet quite low, right ? What is the corresponding FWMH ?

The decoding scores are indeed quite modest (peak of ~0.08, where chance is 0 and perfect prediction is 1*). The effect sizes we are dealing with are therefore quite small. However, given the large number of static flashes and smooth motion sequences presented to participants, our results are highly significant, with sustained decoding at $p < .01$.

*Note, due to a scaling issue the decoding scores have doubled. This only affects the scale of the visualisations in Fig 1 and has no bearing on their statistical significance. For further details see our response to R2C12) below.

R2C7) Figure 2: how do you define evidence ? Is it the log-odd ratio of the predicted probability?

Position evidence is defined as the posterior probability of each class (i.e. angular position). These are generated via the 'predict_proba' function of the LDA classifier in scikit-learn. We now specify this in the Method (Lines 694-696):

'We extracted predicted posterior class probabilities from these pre-trained LDA models (via the 'predict_proba' function in scikit-learn), based off participants' neural responses to the smoothly moving stimuli.'

R2C8) even if it is obvious, define the acronym FWHM

We now define this when first using the acronym (Line 200).

R2C9) l.456: check syntax of "to determine where a stimulus just was"

Thanks for highlighting this awkward phrasing, we have revised as follows (Lines 466-468):

'either to determine the recent position of a stimulus with high-reliability (peak), or the probable present position a stimulus, with lower reliability (centroid).'

R2C10) l. 532 how do you maintain fixation ?

See our response to R1C1) for a comprehensive answer to this question. In short, while we did not employ an eye tracker, we did explicitly instruct participants not to move their eyes from fixation. We also used a fixation target which has shown to best reduce eye movements and promote fixation (Thaler et al., 2013 – *Vision Research*). Given that the stimuli were presented at a distance of 7.7-9.4 dva from fixation (from inner to outer edge), with the static stimuli presented at a rapid temporal rate (1 every 200 ms) and the dynamic stimuli moving at 48 dva/s, it is highly unlikely that participants

would have been able to continually and reliable fixate on the stimuli, nor indeed did they have any good reason to attempt to. Moreover, given the specifics of the decoding analyses we conducted (see R1C1 above), small breaks of fixation will have merely added noise to the signal and cannot have introduced reliable position-related confounds.

R2C11) l. 559 "extract" > "extracted"

Thanks for catching this typo, we have corrected it in the revised manuscript.

R2C12) l. 581 - intuitively, for a perfect match, arg(1) = 0 and the score is 1/2 - could you give more hints as to what is wrong?

We thank the reviewer for their close reading of this. The angular decoding score originally formulated by King and colleagues (2016) is scaled between -pi/2 and pi/2, and our intention was to rescale this to the interval [-1, 1] for the sake of interpretability. However, re-examining our code, we can see that we mistakenly scaled the scores to the interval [-0.5, 0.5] rather than [-1, 1]. Note, this only affects the SVR-based decoding results, and that this only affects the *scale* of the visualisations, not their statistical significance. In the revised manuscript, for the sake of clarity, we have rescaled the results to [-1, 1] and have updated the equation as follows:

$$Decoding\ score\ = \frac{2\left(\frac{\pi}{2} - \frac{1}{n}\sum_{j=1}^{n}\left|\arg\left(\frac{e^{i\widehat{y_j}}}{e^{iy_j}}\right)\right|\right)}{\pi}$$

To follow the Reviewers example, for a perfect match the score now reduces to $\frac{2\left(\frac{\pi}{2}-0\right)}{\pi}$ = 1. For guessing it reduces to $\frac{2\left(\frac{\pi}{2}-\frac{\pi}{2}\right)}{\pi}$ = $\frac{2(0)}{\pi}$ = 0, and for completely opposite prediction it reduces to, $\frac{2\left(\frac{\pi}{2}-\pi\right)}{\pi}$ = $\frac{2\left(-\frac{\pi}{2}\right)}{\pi}$ = -1.

The rescaled empirical data from Fig 1 are plotted below:

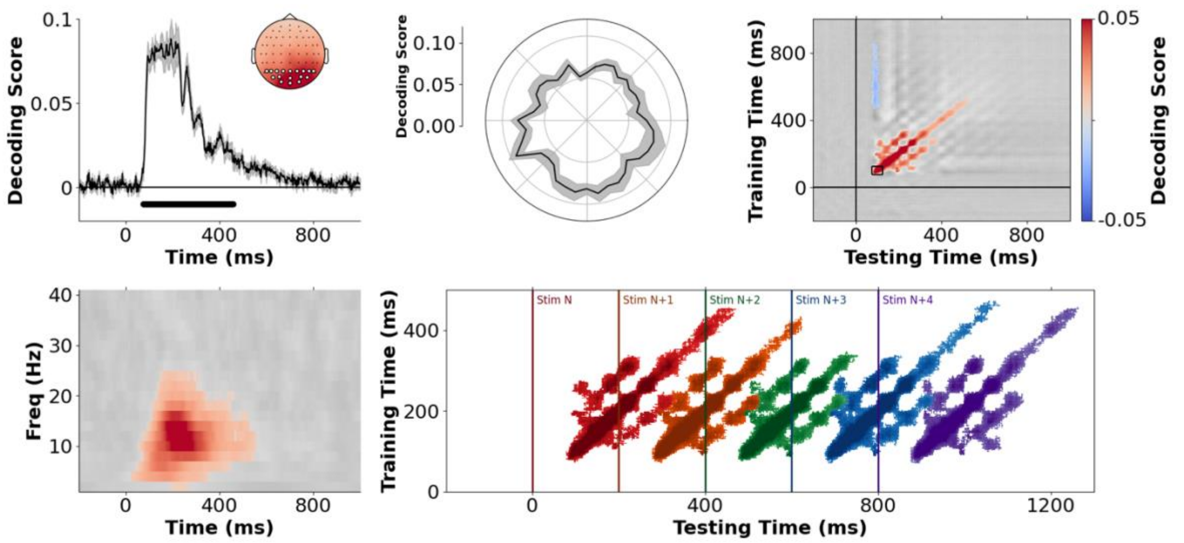

Fig R4. The rescaled empirical data from Fig 1. Note, none of the dynamics, nor the results of the significance tests, have changed. The only difference is that the scaling of decoding scores has doubled.

R2C13) l. 611 : typo in equation: e x p (,) > e x p (.)

Thanks for catching this, we have corrected the equation as follows:

$$\bar{a} = \frac{1}{n}\sum_{j=1}^{n} w_j \cdot \exp(i \cdot a_j)$$

R2C14) the evidence seems to be uniform at all angles, while one might expect it to be less efficient at the midline, where the information has to travel through the corpus callosum. is there experimental evidence for this midline in the motion extrapolation literature?

We thank the reviewer for raising this interesting point. The current data are equivocal as to whether there is an effect of crossing the visual midline, since the main spatiotemporal maps (e.g., Fig 2) are averaged over all angular positions. To assess activity during midline transitions we would need to restrict our focus to 2/40 positions, greatly reducing the SNR of our analyses. As such, proper assessment of this would require further experimentation, in which these transition points are over-sampled, allowing sufficient resolution to properly address this question.

To our knowledge, the only neural data of relevance to this question comes from a preprint by Benvenuti and colleagues (2020). They show evidence that the anticipatory responses of V1 neurons to moving stimuli are abolished when stimuli approach from the ipsilateral visual hemifield. This provides evidence that anticipatory responding is facilitated by intra-hemispheric mechanisms (e.g., lateral connections between neurons).

We now discuss this point in the revised manuscript (Lines 485-491), highlighting it as an interesting avenue for further research:

'… Specifically, studies could examine the potential roles of laterally spreading activity[4,6], suppressive travelling waves[45], and related mechanisms (for a recent review see[2]), in dampening activity at the trailing edge and/or enhancing activity at the leading edge of a moving stimulus, driving a distribution shift. For instance, it may be interesting to examine the degree to which anticipatory effects are abolished during visual midline transitions (i.e. when stimuli move from one hemifield to the other), as laterally spreading activation will be disrupted, with signals instead passing through the corpus callosum (e.g., see[10]).'

References

Benvenuti, G., Chemla, S., Boonman, A., Perrinet, L., Masson, G. S., & Chavane, F. (2020). Anticipatory responses along motion trajectories in awake monkey area V1. *BioRxiv*, 2020-03.

Chemla, S., Reynaud, A., Di Volo, M., Zerlaut, Y., Perrinet, L., Destexhe, A., & Chavane, F. (2019). Suppressive traveling waves shape representations of illusory motion in primary visual cortex of awake primate. *Journal of Neuroscience*, 39(22), 4282-4298.

Eagleman, D. M., & Sejnowski, T. J. (2000). Motion integration and postdiction in visual awareness. *Science*, 287(5460), 2036-2038.

Johnson, P. A., Blom, T., van Gaal, S., Feuerriegel, D., Bode, S., & Hogendoorn, H. (2023). Position representations of moving objects align with real-time position in the early visual response. *eLife*, 12, e82424.

Khoei, M. A., Masson, G. S., & Perrinet, L. U. (2017). The flash-lag effect as a motion-based predictive shift. *PLoS computational biology*, 13(1), e1005068.

King, J. R., Pescetelli, N., & Dehaene, S. (2016). Brain mechanisms underlying the brief maintenance of seen and unseen sensory information. *Neuron*, 92(5), 1122-1134.

King, J. R., & Wyart, V. (2021). The human brain encodes a chronicle of visual events at each instant of time through the multiplexing of traveling waves. *Journal of Neuroscience*, 41(34), 7224-7233.

Hajonides, J. E., Nobre, A. C., van Ede, F., & Stokes, M. G. (2021). Decoding visual colour from scalp electroencephalography measurements. *NeuroImage*, 237, 118030.

Herzog, M. H., Drissi-Daoudi, L., & Doerig, A. (2020). All in good time: long-lasting postdictive effects reveal discrete perception. *Trends in Cognitive Sciences*, 24(10), 826-837.

Maus, G. W., & Nijhawan, R. (2008). Motion extrapolation into the blind spot. *Psychological Science*, 19(11), 1087-1091.

Quax, S. C., Dijkstra, N., van Staveren, M. J., Bosch, S. E., & van Gerven, M. A. (2019). Eye movements explain decodability during perception and cued attention in MEG. *Neuroimage*, 195, 444-453.

Schwartz, G., Taylor, S., Fisher, C., Harris, R., & Berry, M. J. (2007). Synchronized firing among retinal ganglion cells signals motion reversal. *Neuron*, 55(6), 958-969.

Thaler, L., Schütz, A. C., Goodale, M. A., & Gegenfurtner, K. R. (2013). What is the best fixation target? The effect of target shape on stability of fixational eye movements. *Vision research*, 76, 31-42.

Turner, W., Sexton, C., & Hogendoorn, H. (2023). Neural mechanisms of visual motion extrapolation. *Neuroscience & Biobehavioral Reviews*, 105484.

Whitney, D., & Murakami, I. (1998). Latency difference, not spatial extrapolation. *Nature neuroscience*, 1(8), 656-657.

Wolff, M. J., Jochim, J., Akyürek, E. G., & Stokes, M. G. (2017). Dynamic hidden states underlying working-memory-guided behavior. *Nature Neuroscience*, 20(6), 864-871.
