# Supplementary material for: Predictable motion is progressively extrapolated across temporally distinct processing stages in the human visual cortex
Source: PLoS Biol. 2025 May 23;23(5):e3003189. doi: 10.1371/journal.pbio.3003189 (PMC12133177; doi:10.1371/journal.pbio.3003189)
Supplement: Attachment — Submitted filename: Turner_Response_Letter_R2.pdf [file pbio.3003189.s006.pdf]

Response to Reviewers

Thank you for the opportunity to submit a revised version of our manuscript. We are grateful for the constructive feedback provided by both reviewers, and have responded to each of their comments below.

Throughout this document reviewer comments will be bolded and quoted sections from the manuscript will be italicized. For clarity and cross-referencing purposes, we have coded each of the reviewers' comments (e.g., R1C1, R1C2 etc.). Line numbers are used to indicate the location of specific changes in the revised manuscript. Figures which are included in the response letter but not in the revised manuscript are prefixed with the letter R. Figures included in the revised manuscript retain their manuscript-specific figure number. All changes in the revised manuscript have been highlighted in yellow.

Reviewer #1 (Laurent U Perrinet): I would like to thank the authors for the new version of their manuscript, as well as for the detailed response to our comments. These two elements allow me to judge that this article is now acceptable for publication in this journal. I have just a few minor points that could be included in the final version.

We thank Prof. Perrinet for his constructive feedback and positive evaluation of the revised manuscript.

R1C1) The choice of decoding score (point R2C12) can be simplified by giving it a physical meaning. By using a value between -1 and 1, we are in fact calculating the cosine of the difference between the predicted angle and the actual angle. It may be simpler simply to give this angle difference.

Thanks for this interesting suggestion. To get a sense for how our custom decoding score relates to the cosine of the angular difference between the actual and predicted position of the stimulus, we have simulated these two measures across all possible angular differences (Fig R1 below). Interestingly, while these approaches indeed generate outputs which lie in the same range [-1, 1], the custom decoding score varies linearly across angular differences, but the cosine does not. Given this, we feel that our custom decoding score (which is simply a scaled version of the decoding score used by King, Pescetelli, & Dehaene, 2016 – *Neuron*) is the most appropriate measure to employ since it is can be interpreted in an intuitively appealing (linear) manner.

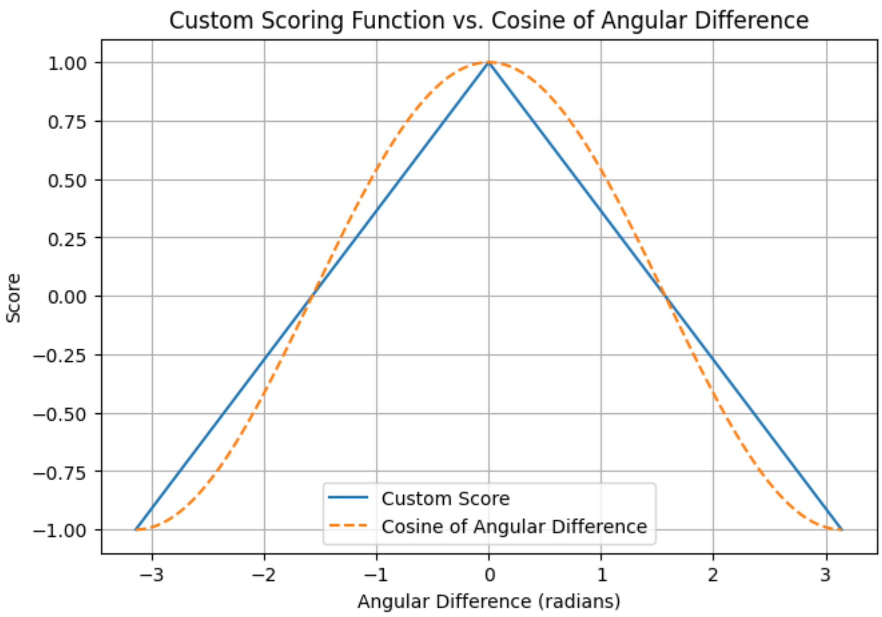

Fig R1. Comparing our custom scoring function to the cosine of the angular difference. The blue line shows outputs of the custom scoring function we employ (y-axis), across a range of angular differences (x-axis). The orange line shows the corresponding cosine of the angular differences.

R1C2) In Fig 2A, you could mention that the "hockey club curve" is similar to that observed in the Frohlich effect (see for instance work from Jancke) - this would also allow you to justify the fact that the temporal resolution here is not enough to conclude for a "jump" at reversal in panel C.

Thanks for this observation. We agree that there are similarities between the 'hockey stick' shaped path which emerges in Fig 2A and the theoretical results of Jancke & Erlhagen (2009) who simulated the emergence of the Fröhlich effect (see their Fig 25.4). We also note that this is in line with predictions from a model by Khoei and colleagues (2017) who simulated the flash-initiated condition of the flash-lag effect, which is at least qualitatively similar to the Fröhlich effect (see their Fig 4A). In the revised manuscript we now discuss the pattern in Fig 2A in relation to these theoretical results as follows (Lines 180-190):

'Fig 2 shows three such maps time-locked to either stimulus onset, stimulus offset or the reversal point in the motion sequences. Examining these, it is clear that the position of the moving stimuli can be tracked from participants' neural response patterns. From ~75ms after stimulus onset position tuning emerges, as evidenced by the high probability region which forms around the initial position of the moving stimulus. The high probability region then shifts, drawing out a 'hockey stick' shaped pattern (reminiscent of observations in existing theoretical models, see[35,36]) and tracks the position of the moving stimulus. After motion reversal (Fig 2C) the high probability region reverses direction and continues to track the new trajectory of the stimulus, although the temporal resolution of the maps is insufficient to evaluate whether an abrupt jump or gradual shift in tracking occurs (see [7, 36] for relevant considerations).'

R1C3) in point R2C7 evidence is defined as the posteriori probability however position evidence (e.g., in Fig2 or 3, 4) can be negative. Please provide a clear definition in the methods.

Thanks for highlighting this potentially confusing point. These values are negative because we calculate ‘position evidence’ by subtracting away chance probability (i.e. the probability of randomly guessing the position of the stimulus, which is 1/40 or 0.025). In the revised manuscript we now clarify this when first referring to ‘position evidence’ in the caption to Fig 2 (Lines 202-203):

‘Red indicates high probability regions and blue indicates low probability regions (‘position evidence’ gives the difference between the posterior probability and chance)’.

Similarly, we further clarify this in the Method (Lines 744-747):

‘In visualising the results of the LDA-based analyses (Figs 2-4), posterior probabilities are converted to ‘position evidence’, a measure of the difference between a given posterior probability value and chance (i.e. the probability of correctly guessing the stimulus’ position, which is 1/40 or 0.025).’

Reviewer #2: I stepped in for R1 (as a new reviewer) after a first round of revision.

We thank the reviewer for taking the time to join this process and for their helpful feedback and suggestions.

R2C1) In my reading, the main concern of R1 is that reported effects may have oculomotor origins rather than visual-processing properties inherent in the visual system. I do agree with the original R1 that it would have been more convincing if the authors had included careful eye-tracking when collecting their data (simply assuming participants follow instructions to fixate may be too optimistic). In response to this main first concern of R1, the authors now make the case that their findings are not driven by muscle artifacts by the eyes because their decoding localizes to posterior electrodes. While I agree this rules out the contribution of muscle artefacts associated with eye movements, it does not necessarily rule out potential secondary consequences of eye movements (moving the visual stimuli over the retina) that may also contribute to the decoding from visual areas. Thus, just because the decoding is posterior, it may still reflect a secondary consequence of eye movements (or micro eye movements), rather than reflecting a computation inherent to the visual system. This could possibly be acknowledged (or provided further evidence against). That said, I find the additional arguments convincing, such as how their decoder was trained on a case with static stimuli, and tested on a case with predictably moving stimuli.

We are glad the reviewer agrees with us that, in this specific analytic context, eye movements cannot be spuriously driving the observed results. We are also grateful that they have highlighted the subtle but important scenario in which secondary effects of eye movements (i.e. changes in visual cortex activity driven by small eye movements and the resulting movement of stimuli across the retina) give rise to confounding effects. We now consider this possibility in the revised manuscript and clarify the aspects of our analyses which allow us to be confident that such a confound cannot explain our findings (Lines 579-589):

'… Finally, for the main LDA-based mapping analyses we restricted our focus to only occipital electrodes, and trained the decoding models only on neural activity patterns 75-125 ms after the onset of the localiser flashes. Since saccade onsets, and the resulting eye-movement-related confounds in decoding performance, typically occur after >200 ms[48], this further reduces the likelihood of eye-movement confounds explaining our results. The fact that we trained on such early timepoints, and generalized models from flash-evoked activity to motion-evoked activity, also reduces concerns about insidious confounding driven by secondary effects of eye movements (e.g., changes in visual cortex activity driven by small eye movements and the resulting movement of stimuli across the retina), as it is unlikely that the static and moving stimuli will have reliably triggered common small eye movement patterns, particularly during such early time windows.'

R2C2) I also agree with R1 that the data presentation could benefit from further statistical substantiation. For example, while figure 3 is extensively described on pages 7-8, there appears no statistical quantification to back things up for this point (and the same appears to apply for figure 2). This remains a bit odd, also to me.

Thanks for highlighting this. As outlined in the previous revision, we intended for the analyses in Fig 3 to be qualitative and exploratory given speculative nature. However, in light of the fact that this concern is shared by both reviewers, we have decided to now also provide quantitative statistical tests of the key patterns we are highlighting.

To this end, we now also extract centroids and peaks from the individual-level probability distributions, allowing us to create sample distributions of these two discrete position estimates. We plot the mean and standard error of these distributions, in addition to the peak and centroid of the group-averaged spatial probability distribution in revised Fig 3 (see below). Note, we have opted to retain the peak and centroid estimates extracted from the group-level distribution, as these have the advantage of being extracted after first averaging over the noise present within the individual-level distributions (in other words, the estimates extracted on the individual level are more affected by noise). We now also plot the standard error of the group averaged probability distributions to help indicate their reliability (see shaded grey regions). Finally, we conduct quantitative statistical tests with the distributions of individual-level peaks and centroids, to confirm that the qualitative patterns we are highlighting are statistically significant. The results of these analyses are reported in the revised manuscript as follows (Lines 225-242):

'Considering Fig 3A&B, an important auxiliary question arises: how might a point-estimate of the stimulus' real-time location be read out from these probability distributions? One option is to take the point of maximum probability (green triangles in Fig 3A&B show the peak of the group-level distribution, green squares and whiskers show the mean and standard error of the distribution of individual-level peaks). However, even after considerable exposure to predictable motion (panel B) this estimate continues to lag the real-time position of the stimulus (marked with the vertical black line). One sample t-tests confirmed that the individual-level peaks significantly lagged the stimulus, both at the start of motion (t = -3.49, p = .003) and mid-motion (t = -2.20, p = .042). An alternative option is to take the centroid (i.e. the vector average), to better leverage information contained within the entire distribution. Interestingly, this yields an estimate (orange triangles) which initially aligns with the distribution peak (panel A, a paired sample t-test found no evidence of a significant difference between the peak and centroid distributions, t = 1.44, p = .17, with a one sample t-test indicating the individual-level centroids significantly lagged the stimulus, t = -4.65, p < .001). After ongoing exposure to predictable motion, this shifts to align with the real-time position of the stimulus (panel B, one-sample t-test found no evidence of a significant difference between centroids and stimulus position, t = 1.13, p = 0.27, paired sample t-test indicates significant difference between peaks and centroids, t = -3.70, p = 0.002).'

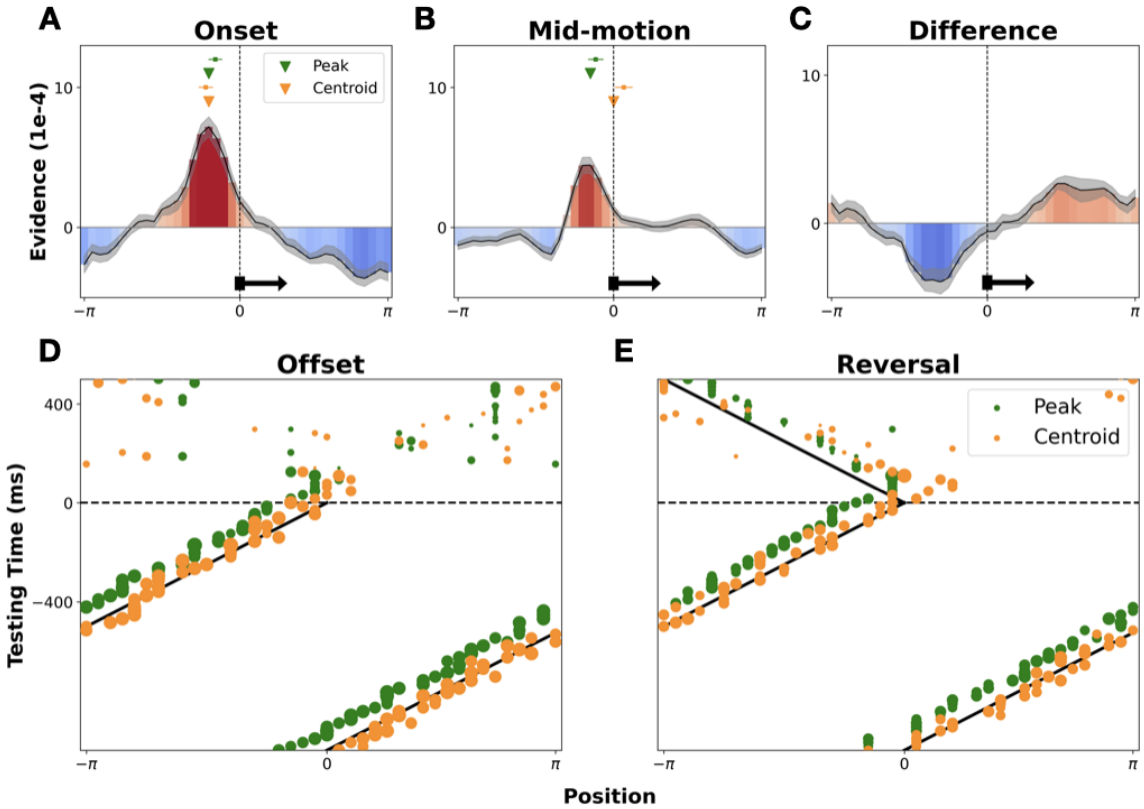

Fig 3. Examining the shape and potential discrete read-out of decoded probability distributions. Panels **A)** and **B)** show group-level averaged time slices through the maps in Fig 2, at motion onset (75-125) and mid-motion (-1000-0 ms) respectively. Shaded grey lines show the standard error of the group-averaged probability distribution. Green inverted triangles show the point of peak probability and orange triangles show the centroid (vector average) of the group-level distribution. The mean and standard error of the individual level centroids and peaks are shown with green/orange squares and whiskers. Panel **C)** shows the difference between the probability distributions extracted from mid-

motion and immediately following stimulus onset. In panels **A-C)** the true position, and direction, of the stimulus is marked by a vertical black dashed line and horizontal black arrow. Panels **D)** and **E)** show timepoint-specific markers of peak probability (green) and centroid (orange), overlaid on the real-time position of the stimulus around stimulus offset and reversal. Peaks and centroids are calculated every 15 ms, with the certainty of the estimate (taken as the peak height or vector average length, respectively) dictating the size of the plotted dot.

We report the details of these new analyses in the method as follows (Lines 756-759):

'To statistically test for differences between the distributions of peaks and centroids extracted from individual-level data, and between these distributions and the true position of the stimulus, we conducted paired sample and one sample t-tests (two-tailed) respectively.'

Finally, we note that a statistically thresholded version of Fig 2 is provided in Supplement 1, meaning that all empirical figures are now accompanied by quantitative statistical testing.

In addition to the above points, R1 made several other good (and critical) comments, but I do not see these as major breaking points, and the authors' responses appear reasonable to me.

We are glad the reviewer found our responses to be reasonable.

As a new reviewer brought in at this stage, reading the work did inevitably prompt some new points that I would like to share. However, I wish to refrain from listing these as major concerns, also because I see as my primarily role to evaluate the revisions done in response to original R1, as I reflected on above. I nonetheless hope my additional comments may be perceived as useful.

Thanks for the additional constructive comments.

R2C3) I was wondering the following about the data in figure 3. Given models were trained in response to localizer stimuli with sudden onsets, is it not trivial that the decoded data differ when applying the model to the onset of the motion (similar to the onset of the localizer) versus mid-motion (that is more distinct from the localizer, given single localizer stimuli were never presented for this long)? This difference (in figure 3) could thus either reflect a change in how predictable motion is represented (as currently interpreted) but may also be driven by an increasing mismatch between localizer and test data (when comparing onset vs. mid-motion) in terms of time from stimulation onset? (It appears to me that to rule this out, the authors would have had to also include long-duration localizer stimuli, and train the decoding model to both early-onset and mid-way points in the localizer data). I also note how the reported difference appears to be largely driven by a change in magnitude (more so than in reconstructed position; given the peaks appears to remain largely unaffected).

We agree that simply observing *a difference* between models tested in contexts similar to those they were trained in (e.g., motion onset) compared to those tested in more novel contexts (e.g., mid-motion) may not always be informative. However, in this specific case we think the difference we observe is valid and informative, for three main reasons. Before outlining these reasons, however, we wish to briefly note that to collect long duration localiser data (as the reviewer suggested) we would need to greatly extend the duration of the experiment (e.g., collecting 1s localisers with no ISI would take 5x as long). Given this, we feel that such an endeavour is beyond the scope of the present study. Below we outline our argument as to why the difference we are observing is valid and informative. Then we outline changes we have made to the manuscript to better consider and clarify this point.

First, the *form* of the difference we observe suggests that it is more than just a trivial artifact of our decoding approach. Specifically, we see an asymmetry which is *conditional on the underlying direction of the stimulus*. Remember that to generate Fig 3 we first flip one of the position maps and then average across both motion directions. As such, any trivial spatial differences (e.g., a general leftwards or rightwards bias) will cancel out. We also see no signs of a similarly trivial difference driven solely by a blurring/widening of the distribution. Importantly, the direction of the asymmetric shift we observe is also opposite to what would be expected if we were simply tapping into later-stage positional representations. Such representations would increasingly lag the stimulus, and thus drive a shift in the opposite direction. Given this, we feel it is reasonable to conclude that the observed asymmetry arises from ongoing exposure to motion.

The second point which argues against the triviality of this finding, comes from consideration of the temporal generalisation analysis in Fig 2E (duplicated below). Specifically, we observe activity dynamics that are predominantly transient and evolving, with a strong diagonal response in the temporal generalization matrix and only brief, transient periods of peri-diagonal generalization. This suggests that decoders trained on early time periods, will not be strongly contaminated by later representations, and thus will act somewhat like ‘filters’ for early position specific activity patterns. The fact that such models successfully generalise to mid-motion activity (albeit with a slight amplitude drop, as noted by the reviewer), suggests that the motion of the stimulus across the visual field evokes effectively equivalent position-specific activity patterns as the localisers, but with an asymmetric shift in the direction of stimulus motion.

Finally, the changes we observe mirror observations made via direct recordings of neural activity in animals – specifically, a damping of activity behind and an enhancement of activity ahead of the moving stimulus (see Turner et al., 2024 for a review). Whilst this does not conclusively indicate that the changes we are observing are driven by these same mechanisms, this correspondence nevertheless helps to build confidence that what we are observing is not just an artifact of the decoding analysis.

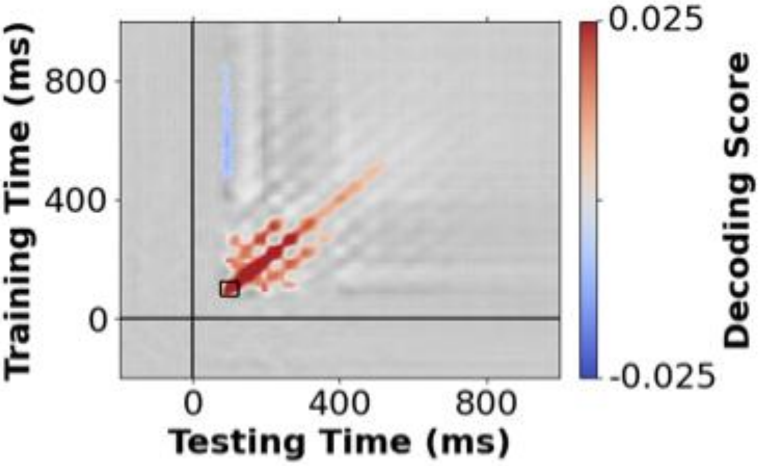

Fig R2. Duplicate of Fig 2E. This figure shows the results of a temporal generalisation analysis in which the group-level performance of timepoint-specific decoders is assessed across all testing time points. The full generalisation matrix is plotted in greyscale with cluster-corrected timepoints overlaid in colour ($p < .01$). The region marked with a black box indicates the training time-period (75-125 ms) for subsequent LDA-based mapping.

In the Discussion section we now consider this point as follows (Lines 511-516):

'… Relatedly, future studies should also attempt to determine the specific neural mechanisms underlying the observed distribution shift. This would help to rule out any concern that this shift trivially arises from the fact that motion onset responses are more similar to localiser evoked activity than mid-motion responses, although this concern is already reduced by the fact that the shift is conditional on the direction of the stimulus, and is consistent with direct observations of neural activity in animals (see [2] for a recent review).'

R2C4) Also, if the primary findings reflect those in figure 4, why do we only see the data for mid-motion in this figure? Would it not be of central interest to also show a temporal profile starting from motion onset to see the development not only with respect to the time after localizer onset (in training data), but also the time after motion onset (in testing data)?

We in fact already show the data that the Reviewer is asking for. This can be found in Supplement 3 of the manuscript where we examine the formation of the predictive shift immediately following motion onset, in both the neural recordings and the network model activity.

However, re-reading the manuscript we can see that we made only limited reference to this supplementary analysis, which is likely why this was not readily apparent to the reviewer. We regret this oversight. To rectify this, in the revised manuscript we now point the reader to Supplement 3, and signpost its content, in multiple sections of the Results.

First, when discussing the main decoding result (Lines 309-314):

'Examining the peak position estimates in Fig 4A&B, we see a sustained forward shift away from the No-Compensation line, with the distance between the points and No-Compensation line increasing over time. This is indicative of sustained extrapolation occurring, leading the encoded position of the stimulus to increasingly deviate from what would be expected as a consequence of neural delays (Supplement 3 shows the formation of this predictive shift immediately following motion onset).'

Similarly, at the end of the caption for Fig 4 (Line 363):

'*See Supplement 3 for an examination of the formation of these effects following motion onset*.'

Finally, after discussing the results of the STDP modelling (Lines 415-416):

'*For an additional exploratory analysis of the timecourse/emergence of this shift see Supplement 3.*'

R2C5) Finally, at various key instances, such as in the title and the abstract, the authors speak of "processing stages". At these key moments, it is not always sufficiently clear whether the authors mean 'brain area in the visual hierarchy' (V1 vs. MT), 'time from motion onset' (onset vs. mid-way), or something else. Upon further reading, it appears these terms actually reflect time after localizer onset and how this time varying localiser location signal predicts the location of a moving stimulus. Translating this operationalization to processing stages and hierarchy appears less trivial than the initial intuition I had when these terms were introduced. I wonder whether this could/should be better positioned up front, to avoid readers potentially feeling misled by the promise set up at the outset of this paper.

Thanks for this suggestion. Re-reading the abstract and introduction we agree that we could perhaps better position what is meant by 'processing stages'. As such, in the abstract we now: 1) clarify that we are indexing activity '*across temporally distinct processing stages*', 2) no longer refer to the effect occurring *'across the hierarchy'*, since we feel that this may inadvertently prompt readers to believe we are directly recording from specific brain areas, and 3) now refer to the main effect as a '*forwards temporal shift*' rather than a '*widespread temporal shift*', since the latter may also inadvertently prompt readers to believe we are mapping activity from specific brain areas. Finally, in the introduction we now more clearly explain the logic of our specific approach (Lines 69-75):

'… As such, an important open question is whether neural motion extrapolation is a multi-stage phenomenon, that occurs during later-stage cortical processing.

To address this question, one path forward lies in using more global measures of neural activity, to concurrently probe distinct stages of visual processing. For example, neural decoders may be trained at different timepoints following stimulus onset to identify and index neural activity patterns associated with distinct stages of processing (e.g., see[19,20]). …'
